# Supplementary material for: Sorted stem/progenitor epithelial cells of pubertal bovine mammary gland present limited potential to reconstitute an organised mammary epithelium after transplantation
Source: PLoS One. 2024 Oct 18;19(10):e0296614. doi: 10.1371/journal.pone.0296614 (PMC11488748; doi:10.1371/journal.pone.0296614)
Supplement: S1 Fig — Hematoxylin and Eosin-stained sections of mammary tissue from 8 weeks-old mouse (top panel) and 17-months old heifers (bottom panel) were viewed using a NanoZoomer. Scale bar left panels = 2 mm; scale bar right panels = 100 μm. (DOCX) [file pone.0296614.s001.docx]

**
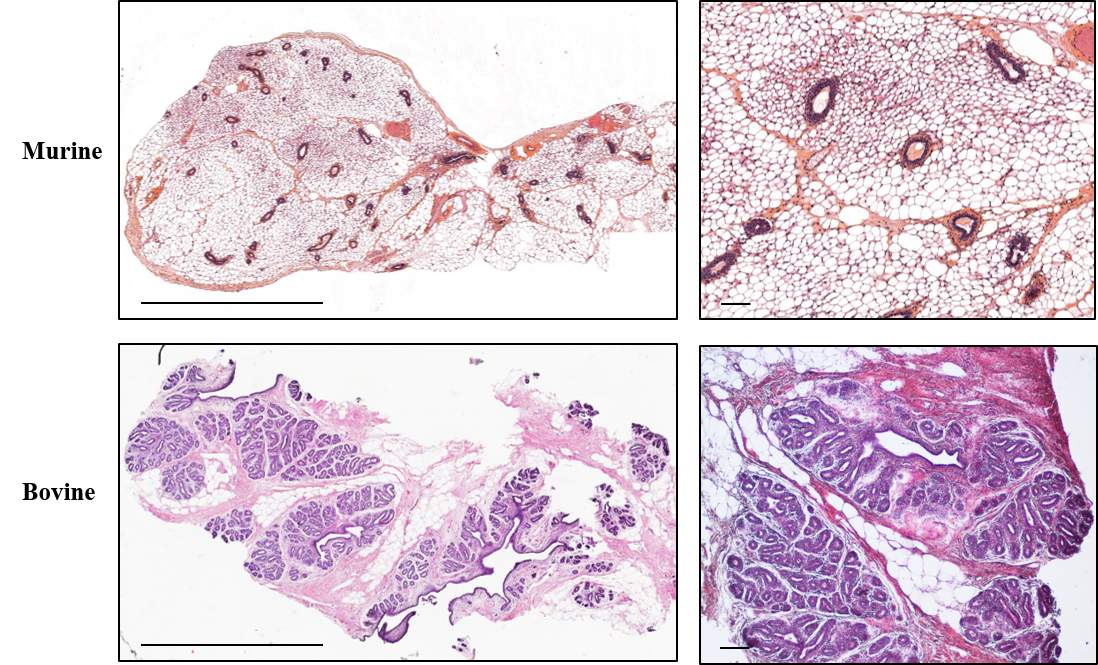
**

**Supplementary Figure S1. Morphology of virgin murine and bovine mammary glands**

Hematoxylin and Eosin-stained sections of mammary tissue from 8 weeks-old mouse (top panel) and 17-months old heifers (bottom panel) were viewed using a NanoZoomer. Scale bar left panels = 2 mm; scale bar right panels = 100 µm.
