# Supplementary material for: Sorted stem/progenitor epithelial cells of pubertal bovine mammary gland present limited potential to reconstitute an organised mammary epithelium after transplantation
Source: PLoS One. 2024 Oct 18;19(10):e0296614. doi: 10.1371/journal.pone.0296614 (PMC11488748; doi:10.1371/journal.pone.0296614)
Supplement: S2 Fig — Bovine primary fibroblasts were isolated from heifer mammary subcutaneous adipose tissue and sub-cultured during 15 passages before their first utilization in the xenotransplantation assay. (A-B) The clonality of the fibroblastic cell culture was analysed based on the expression of fibroblastic markers and the absence of epithelial markers. (A) Fibroblasts at passage 10 were fixed and analysed by indirect immunofluorescence for the basal epithelial cell marker cytokeratin 14 (green), the luminal epithelial cell marker cytokeratin 7 (orange) and the stromal protein collagen type I (red). Note the absence of epithelial cell markers and the prevalence of the fibroblastic collagen type I marker. Nuclei were counterstained with Hoechst 33342 (blue). Scale bar = 25 μm. (B) A protein fraction was prepared from either cultured bovine fibroblasts (bFIB) at passage 10 or bovine mammary gland parenchyma (PAR) and analysed by SDS-PAGE followed by immunoblotting for either the fibroblastic markers vimentin (VIM; Mr. 57 kDa) and smooth muscle actin alpha (αSMA; Mr. 42 kDa) or the epithelial cell markers E-cadherin (CDH1; Mr. 120 kDa) and cytokeratin 19 (KRT 19; Mr. 44 kDa). Molecular mass markers (kDa) are shown on the left. (C) Cultured bovine fibroblasts at passage 10 were fixed and subjected to immunofluorescence using antisera against telomerase (red) and vimentin (green). The telomeric activity is found in fibroblasts nuclei, maintaining their proliferation capacity through passages. Nuclei were counterstained with Hoechst 33342 (blue). Scale bar = 25 μm. (D) Protein fractions were prepared from irradiated bFIB cultured for the indicated days with or without sorted CD49f+ epithelial cells and analysed by SDS-PAGE followed by immunoblotting for PCNA. (E) Doubling time and viability of the cultured bovine fibroblasts at passages 5, 10, 15 and 20. (F) Viability of cultured irradiated bovine fibroblasts over a three-week period. (DOCX) [file pone.0296614.s002.docx]

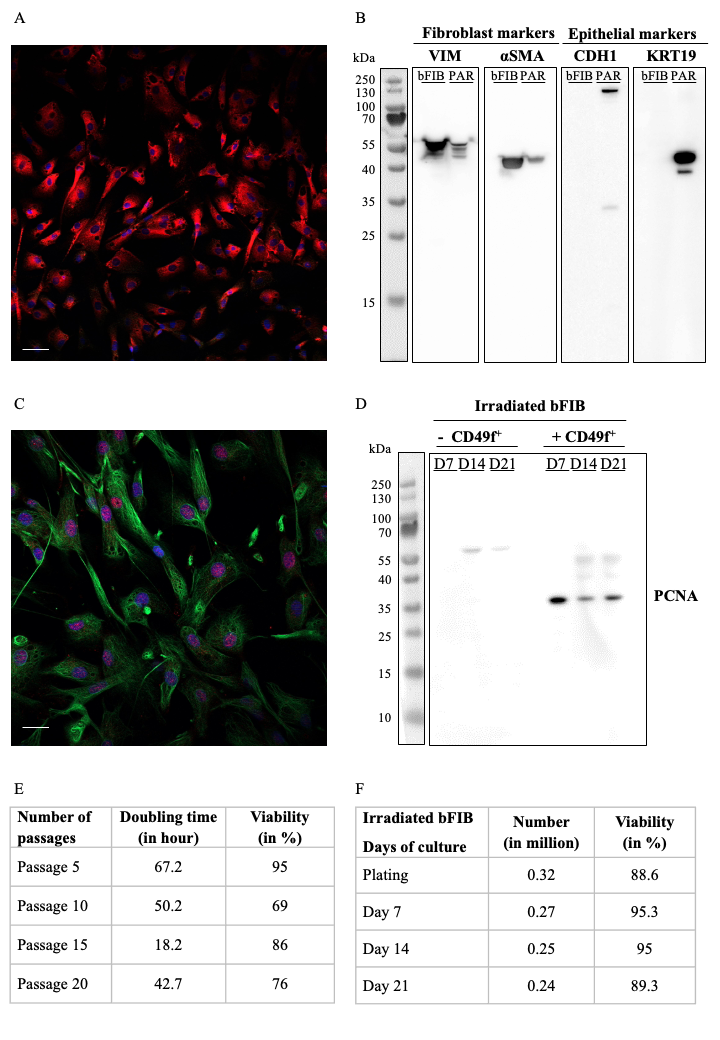


**Supplementary Figure S2. Characterisation of the bovine fibroblasts used for the bovinisation of murine cleared mammary fat pad**

Bovine primary fibroblasts were isolated from heifer mammary subcutaneous adipose tissue and sub-cultured during 15 passages before their first utilization in the xenotransplantation assay. (A-B) The clonality of the fibroblastic cell culture was analysed based on the expression of fibroblastic markers and the absence of epithelial markers. (A) Fibroblasts at passage 10 were fixed and analysed by indirect immunofluorescence for the basal epithelial cell marker cytokeratin 14 (green), the luminal epithelial cell marker cytokeratin 7 (orange) and the stromal protein collagen type I (red). Note the absence of epithelial cell markers and the prevalence of the fibroblastic collagen type I marker. Nuclei were counterstained with Hoechst 33342 (blue). Scale bar = 25 µm. (B) A protein fraction was prepared from either cultured bovine fibroblasts (bFIB) at passage 10 or bovine mammary gland parenchyma (PAR) and analysed by SDS-PAGE followed by immunoblotting for either the fibroblastic markers vimentin (VIM; Mr. 57 kDa) and smooth muscle actin alpha (αSMA; Mr. 42 kDa) or the epithelial cell markers E-cadherin (CDH1; Mr. 120 kDa) and cytokeratin 19 (KRT 19; Mr. 44 kDa). Molecular mass markers (kDa) are shown on the left. (C) Cultured bovine fibroblasts at passage 10 were fixed and subjected to immunofluorescence using antisera against telomerase (red) and vimentin (green). The telomeric activity is found in fibroblasts nuclei, maintaining their proliferation capacity through passages. Nuclei were counterstained with Hoechst 33342 (blue). Scale bar = 25 µm. (D) Protein fractions were prepared from irradiated bFIB cultured for the indicated days with or without sorted CD49f+ epithelial cells and analysed by SDS-PAGE followed by immunoblotting for PCNA. (E) Doubling time and viability of the cultured bovine fibroblasts at passages 5, 10, 15 and 20. (F) Viability of cultured irradiated bovine fibroblasts over a three-week period.
