# Supplementary material for: Sorted stem/progenitor epithelial cells of pubertal bovine mammary gland present limited potential to reconstitute an organised mammary epithelium after transplantation
Source: PLoS One. 2024 Oct 18;19(10):e0296614. doi: 10.1371/journal.pone.0296614 (PMC11488748; doi:10.1371/journal.pone.0296614)
Supplement: S1 Table — (DOCX) [file pone.0296614.s003.docx]

Sorted stem/progenitor epithelial cells of pubertal bovine mammary gland present limited potential to reconstitute an organised mammary epithelium after transplantation

Laurence Finot, Cathy Hue-Beauvais, Etienne Aujean, Fabienne Le Provost, Eric Chanat

PLOS ONE

Finot et al, 2017

**Table S1. Antibodies used for flow cytometry (FACS), Western Blotting and immunofluorescence analyses.**

Finot et al, 2017

| **Primary antibodies / Antigen** | **Host (clone)** | **Manufacturer** | **Reference** | **Dilution (Application)** |
| --- | --- | --- | --- | --- |
| CD24 | CD24-APC, mouse (clone M1/69) | Stem Cell | 60099AZ.1 | 1:10 (FACS) |
| Isotype control | Rat IgG2b-APC | Stem Cell | 60077AZ.1 | 1:10 (FACS) |
| CD49_f_ | CD49_f_-FITC, human and mouse (clone GoH3) | Miltenyi Biotec | 130-097-245 | 1:10 (FACS) |
| Isotype control | Rat IgG2a-FITC | Miltenyi Biotec | 130-102-653 | 1:10 (FACS) |
| Α-Smooth Muscle (αSMA) | Mouse (clone 1A4) | Santa Cruz Biotech | SC32251 | 1:2500 (WB) |
| E-cadherin (CDH1) | Mouse (clone CY-90) | Dako | M3612 | 1:2500 (WB) |
| Bovine Collagen Type I | Rabbit | Merck (Millipore) | AB749P | 1:200 (IF) |
| Cytokeratin 7 (KRT7) | Mouse (clone 5F282) | Santa Cruz Biotech | SC70936 | 1 :100 (IF) |
| Cytokeratin 14 (KRT14) | Goat (clone C-14) | Santa Cruz Biotech | SC17104 | 1 :100 (IF) |
| Cytokeratin 19 (KRT19) | Mouse (clone b170) | Leica Biosystems | NCL-CK19 | 1:2500 (WB) |
| Ki67 | Rabbit | Abcam | Ab15580 | 1:200 (IF) |
| Telomerase reverse transcriptase | Rabbit (clone Y182) | Abcam | Ab32020 | 1:200 (IF) |
| Vimentin | Goat (clone C-20) | Santa Cruz Biotech | SC7557 | 1:2500 (WB) / 1 :100 (IF) |
|  |  |  |  |  |
| **Secondary antibodies** | **conjuguate** | **Manufacturer** | **Reference** | **Dilution (Application)** |
| Goat anti-Mouse | Alexa Fluor 568 | Fisher Scientific | A11031 | 1 :500 (IF) |
| Donkey anti-Goat | Alexa Fluor 488 | Fisher Scientific | A11055 | 1 :500 (IF) |
| Donkey anti-Rabbit | Alexa Fluor 647 | Biolegend | 406414 | 1 :500 (IF) |
| Donkey anti-Rabbit | Alexa Fluor 488 | Fisher Scientific | A11008 | 1 :500 (IF) |
| Rabbit anti-Mouse | HRP | Dako | P0161 | 1 :5000 (WB) |
| Swine anti-Goat | HRP | Invitrogen | G5007 | 1 :5000 (WB) |

Finot et al, 2017

Finot et al, 2017
