# Supplementary figures and images for: Sorted stem/progenitor epithelial cells of pubertal bovine mammary gland present limited potential to reconstitute an organised mammary epithelium after transplantation
Source: PLoS One. 2024 Oct 18;19(10):e0296614. doi: 10.1371/journal.pone.0296614 (PMC11488748; doi:10.1371/journal.pone.0296614)

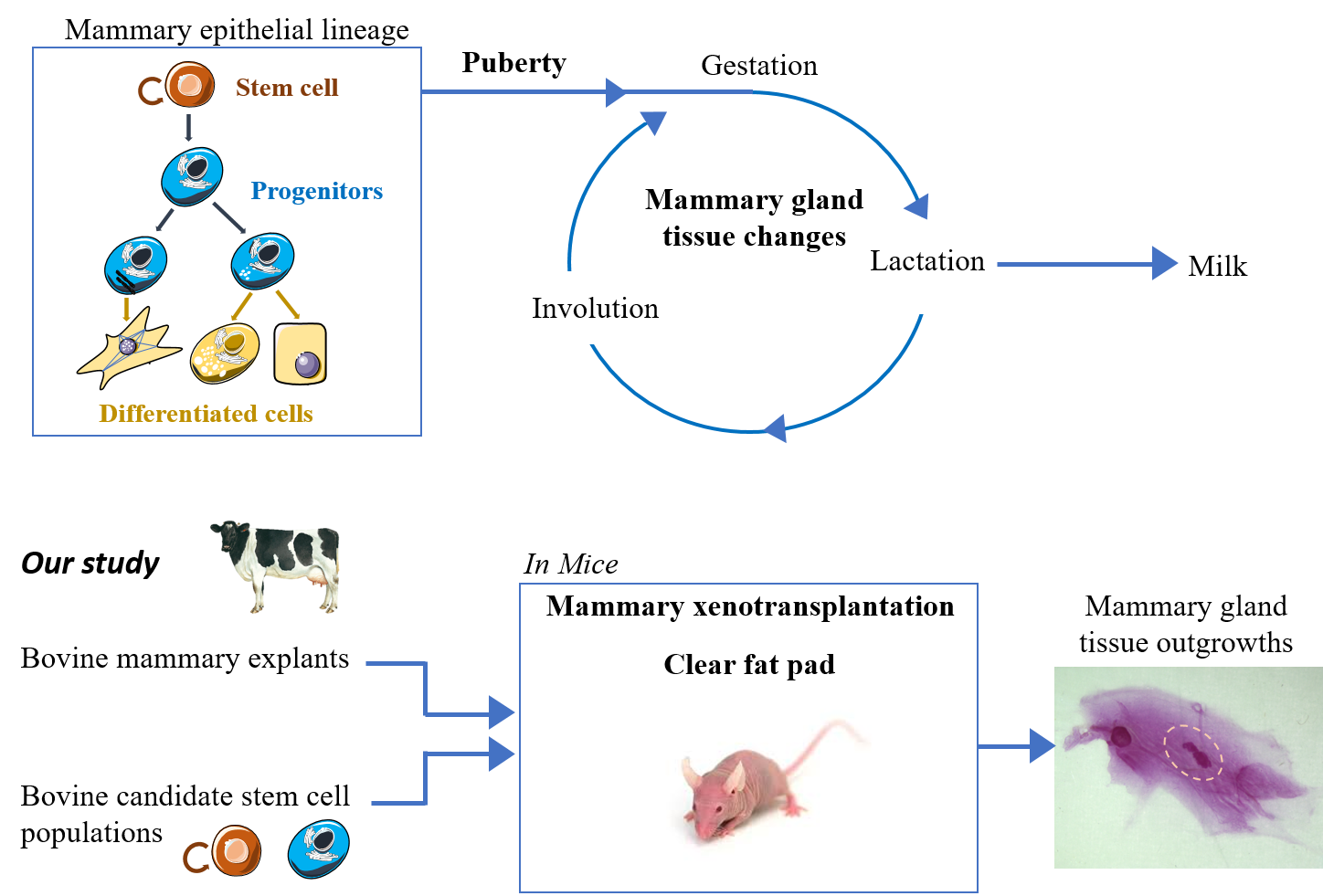

Supplement: S1 Graphical abstract — (TIF) [file pone.0296614.s004.tif]
