# Supplementary material for: Sorted stem/progenitor epithelial cells of pubertal bovine mammary gland present limited potential to reconstitute an organised mammary epithelium after transplantation
Source: PLoS One. 2024 Oct 18;19(10):e0296614. doi: 10.1371/journal.pone.0296614 (PMC11488748; doi:10.1371/journal.pone.0296614)
Supplement: Attachment — Submitted filename: Finot et al PLOS ONE 2024 revision 3 response to Reviewers.docx [file pone.0296614.s006.docx]

Dear Dr. Yi Li,

Following your mail of August 8, 2024 and the helpful comments of Reviewer #3, we are very pleased to re-submitt a revised version of the manuscript in which most suggestions of the Reviewer have been incorporated.

A point-by-point response to the Reviewers' comments could be found below.

We hope that this last version of our manuscript will find your approval.

With best regards.

Response to the reviewer's comments.

Response to General comments:

“Certain figures in the manuscript need some additional data to better support part of the conclusions. The manuscript may also benefit from some language editing. »

As suggested, we have improved images in Fig. 1 and have added a Figure in the Supplementary materials. Regarding the second point, we carried out a thorough rereading of the manuscript and corrected it.

Response to specific points:

“1. In Fig 1, the authors showed the H&E and immunofluorescence staining of the outgrowths generated by xenografted bovine mammary gland explants to demonstrate the structure, and concluded that the outgrowths are highly reminiscent of bovine mammary gland epithelium. To better support this conclusion, a bovine mammary gland control should be included to show the resemblance, and possibly a mouse mammary gland control as well to show the difference. »

We agree and have therefore added images of H&E-stained virgin mouse and cow mammary gland tissues as supplementary Fig.1. The Results and “Materials and Methods” sections have been amended accordingly. Original images were added to the redepository URL.

“2. In Figs 2&3, the authors showed the H&E staining to demonstrate outgrowths without organized epithelial structure generated by sorted bovine epithelial cells. The authors may want to do some immunostaining to confirm the outgrowths are indeed composed of epithelial cells.”

We fully agree that it would have been important to characterise the bovine outgrowths in more detail in order to prove that they are at least partly composed of epithelial cells. Unfortunately, all samples were processed for H&E staining and it is obviously not possible to repeat all these experiments in the time of this review. However, we would like to emphasize that it is doubtful whether the epithelial markers would have exhibited their precise localisation, or even whether they would have been expressed since the outgrowths did not display the typical epithelial organization of mammary gland tissue.

“3. Fig 1B and the middle panel of Fig 1C do not seem very clear when they are magnified. The authors may want to further improve the quality of the pictures.”

As requested, we have improved the quality of Figure 1, notably making the resolution of the images in Fig.1C uniform, with all panels now set to 300 DPI.

“4. In Fig 2C, the authors included “fibroblast only” as control. It might be of interest to include a “no fibroblast” control as well.”

We did not test the transplantation in the absence of bovine fibroblast. The Reviewer is right that this might have been a standard control to do. However, we designed our procedure based on the literature on bovine epithelial cell xenotransplantation in which “bovinisation” of the mouse mammary gland appears to be a prerequisite for successful transplant development. We therefore omitted this control. It was also a matter of reducing the number of tests and controls, which were already numerous, and of reducing the number of mice sacrificed during this experimentation.

“5. The language of the manuscript may need some improvement. & 6. There are some grammar issues and typos in the manuscript. For example, in line 63 “as a results” should be “as a result”, and in line 403 “we though that” probably should be “we thought that”.”

The whole MS was thoroughly edited. We corrected the indicated mistakes as well as some others.
